# Bis(μ-disulfur dinitrido)bis­[diphenyl­tin(IV)]

**DOI:** 10.1107/S1600536808008957

**Published:** 2008-04-10

**Authors:** Alasdair P. M. Robertson, Alexandra M. Z. Slawin, J. Derek Woollins

**Affiliations:** a Department of Chemistry, University of St Andrews, St Andrews, KY16 9ST, Scotland

## Abstract

The title compound, [Sn_2_(C_6_H_5_)_4_(N_2_S_2_)_2_], exists as a centrosymmetric binuclear dimer with the Sn^IV^ centres in distorted trigonal bipyramidal geometry and a central Sn_2_N_2_ core.

## Related literature

For related literature, see: Aucott *et al.* (2002 ▶, 2003 ▶); Bates *et al.* (1986 ▶); Chivers *et al.* (1986 ▶); Jones *et al.* (1985*a* ▶,*b* ▶, 1986 ▶, 1987 ▶, 1988 ▶); Kelly & Woollins (1986 ▶); Read *et al.* (2007 ▶); Slawin & Woollins (2006 ▶).
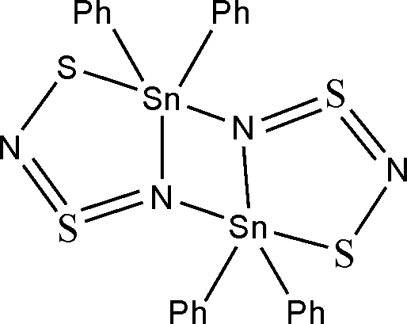


## Experimental

### 

#### Crystal data

[Sn_2_(C_6_H_5_)_4_(N_2_S_2_)_2_]
*M* *_r_* = 730.06
Triclinic, 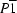

*a* = 8.9235 (6) Å
*b* = 9.2285 (9) Å
*c* = 9.5881 (8) Å
α = 63.809 (2)°
β = 67.309 (2)°
γ = 70.471 (2)°
*V* = 640.72 (9) Å^3^
*Z* = 1
Mo *K*α radiation
μ = 2.30 mm^−1^
*T* = 93 (2) K
0.20 × 0.03 × 0.03 mm

#### Data collection

Rigaku Mercury diffractometer
Absorption correction: multi-scan (*CrystalClear*; Rigaku 2004 ▶) *T*_min_ = 0.923, *T*_max_ = 0.941
4110 measured reflections
2267 independent reflections
2110 reflections with *I* > 2σ(*I*)
*R*_int_ = 0.046

#### Refinement

*R*[*F* ^2^ > 2σ(*F* ^2^)] = 0.035
*wR*(*F* ^2^) = 0.099
*S* = 1.14
2267 reflections
155 parameters
H-atom parameters constrained
Δρ_max_ = 0.85 e Å^−3^
Δρ_min_ = −1.19 e Å^−3^

### 

Data collection: *CrystalClear* (Rigaku, 2004 ▶); cell refinement: *CrystalClear*; data reduction: *CrystalClear*; program(s) used to solve structure: *SHELXS97* (Sheldrick, 2008 ▶); program(s) used to refine structure: *SHELXL97* (Sheldrick, 2008 ▶); molecular graphics: *SHELXTL* (Sheldrick, 2008 ▶); software used to prepare material for publication: *SHELXTL*.

## Supplementary Material

Crystal structure: contains datablocks I, global. DOI: 10.1107/S1600536808008957/bt2692sup1.cif

Structure factors: contains datablocks I. DOI: 10.1107/S1600536808008957/bt2692Isup2.hkl

Additional supplementary materials:  crystallographic information; 3D view; checkCIF report

## Figures and Tables

**Table:** 

Sn1—N1	2.137 (4)
Sn1—N1^i^	2.296 (3)
Sn1—S2	2.5967 (12)

**Table:** 

N1—Sn1—N1^i^	72.82 (15)
N1—Sn1—S2	80.65 (9)
S1—N1—Sn1	121.6 (2)
Sn1—N1—Sn1^i^	107.18 (15)
N2—S2—Sn1	101.63 (14)

Symmetry code: (i) 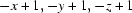.

**Fig. 1.**
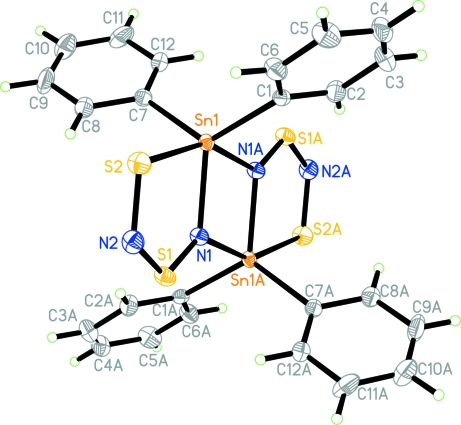
The structure of title compound with displacement ellipsoids drawn at the 50% probability level. Symmetry operator for generating equivalent atoms: (A) 1-x, 1-y, 1-z.

## supplementary crystallographic information

### Comment

The disulfurdinitride dianion is unknown in simple salts but can be isolated in metal complexes (Kelly and Woollins 1986, Jones *et al.* 1985*a*,*b*; Bates *et al.* 1986, Read *et al.* 2007) which may be protonated at the metal coordinated nitrogen (Jones *et al.* 1986, 1988) and we have previously commented on the structural consequences of this protonation (Jones *et al.* 1987). Recently, we developed a new route to disulfurdinitrido complexes from Bu_2_SnS_2_N_2_ (Aucott *et al.* 2002) and examined the metallation of the IrS_2_N_2_ and CoS_2_N_2_ rings using the AuP*R*_3_ cation as a species which is isolobal with a proton (Aucott *et al.* 2003, Slawin and Woollins 2006).

The structure of the title compound  contains tin centres in distorted trigonal bipyramidal geometry and a central Sn_2_N_2_ ring (Figure 1). The binuclaear dimer is disposed about a centre of symmetry. The central core (excluding the phenyl rings) is planar with a mean deviation of 0.01 Å and a maximum deviation of 0.025 Å for N(1). The geometry is very similar to that of [*n-*Bu_2_SnS_2_N_2_]_2_ (Aucott *et al.* 2002). Comparison of the S—N bond lengths with platinum phosphine substituted complexes containing the S_2_N_2_ group reveals that the S—N bond lengths have a different motif to the PMe_2_Ph complex (Jones *et al *1988) and one of the published PPh_3_ complexes (Chivers *et al.* 1986), but are comparable with most others systems containing the disulfurdinitrido anion (Jones *et al.* 1985*a*, Bates *et al *1986).

### Experimental

Ammonia gas (400 ml) was condensed into a dry-ice/acetone cooled Schlenk flask.[S_4_N_3_]Cl (18.47 g, 0.019 moles) was then added, forming a dark red solution. After stirring for 30 minutes, Ph_2_SnCl_2_ (1.68 g, 4.88 mmoles) was added, and the mixture stirred at 195 K for 4 h, before removal of the lower cooling bath, allowing NH_3_(*l*) reflux, and eventually evaporation overnight. The solid products were transferred to a Sohxlet apparatus, containing dry, degassed petroleum ether (140 ml) and cycled for 4 h, by which point the extracts appeared almost colourless. The lower flask was then placed under N2(*g*) at 250 K for 12 h, yielding bright yellow-orange crystals of Ph_2_SnS_2_N_2_, collected by filtration under N_2_. Yield: 0.092 g, 5.15%. IR Spectrum (KBr Pellet, cm^-1^): 3063 (*m*), 2963 (*m*), 1428 (*versus*), 1070 (*s*), 1024 (*s*), 899 (*s*), 729 (*s*), 694 (*s*), 636(*s*), 440 (*s*) and 382 (*s*), ^1^H NMR: δH 7.56–7.53 (4*H*, m, Ph) and 7.40–7.37 (6*H*, m, Ph), Mass Spectrum: EI m/*z* (%): 366.05 (Ph_2_SnS_2_N_2_, 5), 288.99 (PhSnS_2_N_2_, 2), 257.01 (PhSnSN_2_, 2), 197.01 (PhSn, 68), 77.06 (Ph, 25) and 63.96 (S2, 8), Melting Point: 411–13 K.

### Refinement

All H atoms were included in calculated positions (C—H distance 0.95Å) and were refined as riding atoms with U_iso_(H) = 1.2 U_eq_(parent atom, and aryl H atoms). The highest peak in the difference map is 0.95 Å from atom S(2) and the deepest hole is 0.96 Å from Sn(1)

### Crystal data

**Table:**

[Sn_2_(C_6_H_5_)_4_(N_2_S_2_)_2_]	*Z* = 1
*M**_r_* = 730.06	*F*_000_ = 356
Triclinic, *P*1	*D*_x_ = 1.892 Mg m^−^^3^
*a* = 8.9235 (6) Å	Mo *K*α radiation λ = 0.71073 Å
*b* = 9.2285 (9) Å	Cell parameters from 2584 reflections
*c* = 9.5881 (8) Å	θ = 2.5–28.3º
α = 63.809 (2)º	µ = 2.30 mm^−^^1^
β = 67.309 (2)º	*T* = 93 (2) K
γ = 70.471 (2)º	Prism, yellow
*V* = 640.72 (9) Å^3^	0.20 × 0.03 × 0.03 mm

### Data collection

**Table:**

Rigaku Mercury diffractometer	2267 independent reflections
Radiation source: rotating anode	2110 reflections with *I* > 2σ(*I*)
Monochromator: confocal	*R*_int_ = 0.046
Detector resolution: 0.83 pixels mm^-1^	θ_max_ = 25.1º
*T* = 93(2) K	θ_min_ = 2.5º
ω and φ scans	*h* = −9→10
Absorption correction: multi-scan(CrystalClear; Rigaku 2004)	*k* = −7→11
*T*_min_ = 0.923, *T*_max_ = 0.941	*l* = −8→11
4110 measured reflections	

### Refinement

**Table:**

Refinement on *F*^2^	Secondary atom site location: difference Fourier map
Least-squares matrix: full	Hydrogen site location: inferred from neighbouring sites
*R*[*F*^2^ > 2σ(*F*^2^)] = 0.036	H-atom parameters constrained
*wR*(*F*^2^) = 0.099	*w* = 1/[σ^2^(*F*_o_^2^) + (0.0548*P*)^2^] where *P* = (*F*_o_^2^ + 2*F*_c_^2^)/3
*S* = 1.14	(Δ/σ)_max_ = 0.001
2267 reflections	Δρ_max_ = 0.85 e Å^−^^3^
155 parameters	Δρ_min_ = −1.19 e Å^−^^3^
Primary atom site location: structure-invariant direct methods	Extinction correction: none

### Special details

**Table:**

Geometry. All e.s.d.'s (except the e.s.d. in the dihedral angle between two l.s. planes) are estimated using the full covariance matrix. The cell e.s.d.'s are taken into account individually in the estimation of e.s.d.'s in distances, angles and torsion angles; correlations between e.s.d.'s in cell parameters are only used when they are defined by crystal symmetry. An approximate (isotropic) treatment of cell e.s.d.'s is used for estimating e.s.d.'s involving l.s. planes.
Refinement. Refinement of *F*^2^ against ALL reflections. The weighted *R*-factor *wR* and goodness of fit *S* are based on *F*^2^, conventional *R*-factors *R* are based on *F*, with *F* set to zero for negative *F*^2^. The threshold expression of *F*^2^ > σ(*F*^2^) is used only for calculating *R*-factors(gt) *etc*. and is not relevant to the choice of reflections for refinement. *R*-factors based on *F*^2^ are statistically about twice as large as those based on *F*, and *R*- factors based on ALL data will be even larger.

### Fractional atomic coordinates and isotropic or equivalent isotropic displacement parameters (Å^2^)

**Table:**

	*x*	*y*	*z*	*U*_iso_*/*U*_eq_	
Sn1	0.42885 (3)	0.54581 (3)	0.68014 (3)	0.01479 (16)	
N1	0.6474 (5)	0.4511 (4)	0.5243 (4)	0.0163 (8)	
S1	0.81765 (14)	0.40140 (14)	0.55466 (14)	0.0202 (3)	
N2	0.8196 (5)	0.4231 (5)	0.7071 (5)	0.0229 (9)	
S2	0.64354 (15)	0.49589 (15)	0.82590 (15)	0.0237 (3)	
C1	0.3401 (5)	0.8035 (5)	0.6353 (5)	0.0158 (9)	
C2	0.2544 (6)	0.9079 (5)	0.5220 (6)	0.0238 (11)	
H2A	0.2361	0.8658	0.4569	0.029*	
C3	0.1945 (6)	1.0741 (6)	0.5022 (7)	0.0288 (12)	
H3A	0.1352	1.1441	0.4246	0.035*	
C4	0.2214 (6)	1.1370 (6)	0.5952 (7)	0.0274 (12)	
H4A	0.1820	1.2503	0.5811	0.033*	
C5	0.3050 (7)	1.0347 (6)	0.7075 (7)	0.0301 (13)	
H5A	0.3227	1.0775	0.7725	0.036*	
C6	0.3644 (6)	0.8701 (6)	0.7283 (6)	0.0251 (11)	
H6A	0.4226	0.8013	0.8071	0.030*	
C7	0.2886 (6)	0.3648 (5)	0.8626 (6)	0.0176 (10)	
C8	0.3637 (6)	0.2265 (6)	0.9698 (6)	0.0234 (11)	
H8A	0.4760	0.2148	0.9628	0.028*	
C9	0.2755 (7)	0.1059 (6)	1.0865 (6)	0.0351 (13)	
H9A	0.3265	0.0130	1.1612	0.042*	
C10	0.1130 (7)	0.1206 (7)	1.0945 (7)	0.0346 (13)	
H10A	0.0540	0.0359	1.1725	0.042*	
C11	0.0361 (7)	0.2576 (7)	0.9900 (7)	0.0360 (14)	
H11A	−0.0765	0.2697	0.9976	0.043*	
C12	0.1259 (6)	0.3773 (6)	0.8739 (6)	0.0241 (11)	
H12A	0.0742	0.4705	0.8000	0.029*	

### Atomic displacement parameters (Å^2^)

**Table:**

	*U*^11^	*U*^22^	*U*^33^	*U*^12^	*U*^13^	*U*^23^
Sn1	0.0162 (2)	0.0132 (2)	0.0155 (2)	−0.00201 (15)	−0.00536 (16)	−0.00553 (16)
N1	0.0150 (19)	0.0190 (19)	0.018 (2)	−0.0018 (15)	−0.0066 (16)	−0.0089 (16)
S1	0.0152 (6)	0.0246 (6)	0.0244 (7)	−0.0011 (5)	−0.0084 (5)	−0.0115 (5)
N2	0.022 (2)	0.026 (2)	0.024 (2)	−0.0046 (17)	−0.0108 (18)	−0.0087 (18)
S2	0.0237 (7)	0.0309 (7)	0.0229 (7)	−0.0028 (5)	−0.0107 (5)	−0.0135 (6)
C1	0.015 (2)	0.014 (2)	0.017 (2)	−0.0048 (17)	−0.0007 (19)	−0.0066 (18)
C2	0.032 (3)	0.020 (2)	0.020 (3)	−0.005 (2)	−0.008 (2)	−0.008 (2)
C3	0.029 (3)	0.021 (3)	0.034 (3)	0.002 (2)	−0.014 (2)	−0.008 (2)
C4	0.030 (3)	0.014 (2)	0.035 (3)	−0.005 (2)	−0.007 (2)	−0.007 (2)
C5	0.034 (3)	0.030 (3)	0.038 (3)	−0.005 (2)	−0.012 (3)	−0.021 (3)
C6	0.026 (3)	0.022 (3)	0.028 (3)	−0.001 (2)	−0.009 (2)	−0.011 (2)
C7	0.022 (3)	0.015 (2)	0.017 (2)	−0.0024 (18)	−0.0050 (19)	−0.0074 (19)
C8	0.024 (3)	0.023 (3)	0.021 (3)	−0.005 (2)	−0.007 (2)	−0.005 (2)
C9	0.049 (4)	0.025 (3)	0.020 (3)	−0.009 (2)	−0.010 (3)	0.003 (2)
C10	0.038 (3)	0.037 (3)	0.025 (3)	−0.018 (3)	−0.006 (3)	−0.002 (2)
C11	0.032 (3)	0.046 (3)	0.023 (3)	−0.018 (3)	0.002 (2)	−0.007 (3)
C12	0.025 (3)	0.023 (2)	0.018 (3)	−0.002 (2)	−0.006 (2)	−0.004 (2)

### Geometric parameters (Å, °)

**Table:**

Sn1—C7	2.132 (5)	C4—H4A	0.9500
Sn1—N1	2.137 (4)	C5—C6	1.380 (7)
Sn1—C1	2.138 (4)	C5—H5A	0.9500
Sn1—N1^i^	2.296 (3)	C6—H6A	0.9500
Sn1—S2	2.5967 (12)	C7—C12	1.382 (7)
N1—S1	1.536 (4)	C7—C8	1.392 (6)
N1—Sn1^i^	2.296 (3)	C8—C9	1.385 (7)
S1—N2	1.567 (4)	C8—H8A	0.9500
N2—S2	1.675 (4)	C9—C10	1.385 (8)
C1—C2	1.385 (6)	C9—H9A	0.9500
C1—C6	1.394 (6)	C10—C11	1.381 (8)
C2—C3	1.396 (6)	C10—H10A	0.9500
C2—H2A	0.9500	C11—C12	1.385 (7)
C3—C4	1.379 (7)	C11—H11A	0.9500
C3—H3A	0.9500	C12—H12A	0.9500
C4—C5	1.364 (8)		
			
C7—Sn1—N1	114.01 (15)	C5—C4—H4A	120.3
C7—Sn1—C1	122.65 (16)	C3—C4—H4A	120.3
N1—Sn1—C1	122.73 (15)	C4—C5—C6	121.0 (5)
C7—Sn1—N1^i^	93.55 (15)	C4—C5—H5A	119.5
N1—Sn1—N1^i^	72.82 (15)	C6—C5—H5A	119.5
C1—Sn1—N1^i^	95.25 (15)	C5—C6—C1	120.9 (5)
C7—Sn1—S2	98.61 (12)	C5—C6—H6A	119.6
N1—Sn1—S2	80.65 (9)	C1—C6—H6A	119.6
C1—Sn1—S2	97.87 (12)	C12—C7—C8	118.6 (4)
N1^i^—Sn1—S2	153.42 (10)	C12—C7—Sn1	121.8 (3)
S1—N1—Sn1	121.6 (2)	C8—C7—Sn1	119.5 (3)
S1—N1—Sn1^i^	131.2 (2)	C9—C8—C7	120.3 (5)
Sn1—N1—Sn1^i^	107.18 (15)	C9—C8—H8A	119.9
N1—S1—N2	115.8 (2)	C7—C8—H8A	119.9
S1—N2—S2	120.4 (2)	C8—C9—C10	120.0 (5)
N2—S2—Sn1	101.63 (14)	C8—C9—H9A	120.0
C2—C1—C6	117.8 (4)	C10—C9—H9A	120.0
C2—C1—Sn1	123.6 (3)	C11—C10—C9	120.5 (5)
C6—C1—Sn1	118.6 (3)	C11—C10—H10A	119.8
C1—C2—C3	120.9 (4)	C9—C10—H10A	119.8
C1—C2—H2A	119.6	C10—C11—C12	118.8 (5)
C3—C2—H2A	119.6	C10—C11—H11A	120.6
C4—C3—C2	120.1 (5)	C12—C11—H11A	120.6
C4—C3—H3A	120.0	C7—C12—C11	121.8 (4)
C2—C3—H3A	120.0	C7—C12—H12A	119.1
C5—C4—C3	119.4 (5)	C11—C12—H12A	119.1
			
C7—Sn1—N1—S1	−96.1 (3)	C6—C1—C2—C3	−0.1 (7)
C1—Sn1—N1—S1	92.6 (3)	Sn1—C1—C2—C3	177.8 (4)
N1^i^—Sn1—N1—S1	177.8 (3)	C1—C2—C3—C4	0.5 (8)
S2—Sn1—N1—S1	−0.7 (2)	C2—C3—C4—C5	−0.8 (8)
C7—Sn1—N1—Sn1^i^	86.17 (18)	C3—C4—C5—C6	0.6 (8)
C1—Sn1—N1—Sn1^i^	−85.12 (19)	C4—C5—C6—C1	−0.2 (8)
N1^i^—Sn1—N1—Sn1^i^	0.001 (2)	C2—C1—C6—C5	−0.1 (7)
S2—Sn1—N1—Sn1^i^	−178.51 (14)	Sn1—C1—C6—C5	−178.0 (4)
Sn1—N1—S1—N2	0.6 (3)	N1—Sn1—C7—C12	−120.4 (4)
Sn1^i^—N1—S1—N2	177.8 (2)	C1—Sn1—C7—C12	50.9 (4)
N1—S1—N2—S2	0.2 (4)	N1^i^—Sn1—C7—C12	−47.6 (4)
S1—N2—S2—Sn1	−0.6 (3)	S2—Sn1—C7—C12	156.1 (3)
C7—Sn1—S2—N2	113.77 (18)	N1—Sn1—C7—C8	57.0 (4)
N1—Sn1—S2—N2	0.68 (16)	C1—Sn1—C7—C8	−131.7 (4)
C1—Sn1—S2—N2	−121.35 (18)	N1^i^—Sn1—C7—C8	129.7 (4)
N1^i^—Sn1—S2—N2	−2.5 (3)	S2—Sn1—C7—C8	−26.6 (4)
C7—Sn1—C1—C2	−92.6 (4)	C12—C7—C8—C9	−1.0 (7)
N1—Sn1—C1—C2	77.9 (4)	Sn1—C7—C8—C9	−178.5 (4)
N1^i^—Sn1—C1—C2	5.0 (4)	C7—C8—C9—C10	1.6 (8)
S2—Sn1—C1—C2	161.8 (4)	C8—C9—C10—C11	−2.1 (9)
C7—Sn1—C1—C6	85.2 (4)	C9—C10—C11—C12	2.0 (9)
N1—Sn1—C1—C6	−104.3 (4)	C8—C7—C12—C11	1.0 (7)
N1^i^—Sn1—C1—C6	−177.2 (4)	Sn1—C7—C12—C11	178.4 (4)
S2—Sn1—C1—C6	−20.4 (4)	C10—C11—C12—C7	−1.5 (8)

Symmetry codes: (i) −*x*+1, −*y*+1, −*z*+1.
